# Enzymatic Kinetic Properties of the Lactate Dehydrogenase Isoenzyme C_4_ of the Plateau Pika (*Ochotona curzoniae*)

**DOI:** 10.3390/ijms17010039

**Published:** 2016-01-07

**Authors:** Yang Wang, Lian Wei, Dengbang Wei, Xiao Li, Lina Xu, Linna Wei

**Affiliations:** Research Center for High Altitude Medicine, Qinghai University, Xining 810016, China; yangwangmd@163.com (Y.W.); weilian5318@163.com (L.W.); lixiao2234566@163.com (X.L.); xu020916@163.com (L.X.); weilinnanana@163.com (L.W.)

**Keywords:** testis-specific lactate dehydrogenase (LDH-C_4_), enzymatic kinetics, plateau pika (*Ochotona curzoniae*), hypoxia, Qinghai-Tibet Plateau

## Abstract

Testis-specific lactate dehydrogenase (LDH-C_4_) is one of the lactate dehydrogenase (LDH) isozymes that catalyze the terminal reaction of pyruvate to lactate in the glycolytic pathway. LDH-C_4_ in mammals was previously thought to be expressed only in spermatozoa and testis and not in other tissues. Plateau pika (*Ochotona curzoniae*) belongs to the genus *Ochotona* of the *Ochotonidea* family. It is a hypoxia-tolerant species living in remote mountain areas at altitudes of 3000–5000 m above sea level on the Qinghai-Tibet Plateau. Surprisingly, *Ldh-c* is expressed not only in its testis and sperm, but also in somatic tissues of plateau pika. To shed light on the function of LDH-C_4_ in somatic cells, *Ldh-a*, *Ldh-b*, and *Ldh-c* of plateau pika were subcloned into bacterial expression vectors. The pure enzymes of Lactate Dehydrogenase A_4_ (LDH-A_4_), Lactate Dehydrogenase B_4_ (LDH-B_4_), and LDH-C_4_ were prepared by a series of expression and purification processes, and the three enzymes were identified by the method of sodium dodecyl sulfate polyacrylamide gel electrophoresis (SDS-PAGE) and native polyacrylamide gel electrophoresis (PAGE). The enzymatic kinetics properties of these enzymes were studied by Lineweaver-Burk double-reciprocal plots. The results showed the Michaelis constant (*K*m) of LDH-C_4_ for pyruvate and lactate was 0.052 and 4.934 mmol/L, respectively, with an approximate 90 times higher affinity of LDH-C_4_ for pyruvate than for lactate. At relatively high concentrations of lactate, the inhibition constant (*K*i) of the LDH isoenzymes varied: LDH-A_4_ (*K*i = 26.900 mmol/L), LDH-B_4_ (*K*i = 23.800 mmol/L), and LDH-C_4_ (*K*i = 65.500 mmol/L). These data suggest that inhibition of lactate by LDH-A_4_ and LDH-B_4_ were stronger than LDH-C_4_. In light of the enzymatic kinetics properties, we suggest that the plateau pika can reduce reliance on oxygen supply and enhance its adaptation to the hypoxic environments due to increased anaerobic glycolysis by LDH-C_4_.

## 1. Introduction

The Qinghai-Tibet Plateau, with an average elevation of over 3000 m above sea level, is the highest and also largest plateau on earth and is characterized by extremely harsh environmental conditions such as hypoxia, strong ultraviolet radiation, and cold temperature which expectedly have profound effects on human and animal survival. Over long-term adaption and evolution, both plateau-native humans and animals have developed a series of strategies to adapt to the plateau environment. Also, inhabitants have developed some unique features to avoid disadvantageous environmental aspects and effectively obtain material or energy from their survival environment as to ensure their normal growth and breeding.

Plateau pika (*Ochotona curzoniae*), which is a member of the genus *Ochotona* of the *Ochotonidae* family, is a small, non-hibernating rodent that lives in remote mountain areas at an elevation of 3000–5000 m on the Qinghai-Tibet Plateau. The pika plays an important role in biodiversity of the ecosystem on the plateau and is regarded as a key species since ancient times [1,2]. Fossil samples of pika that are nearly 37 million years old were found by archaeologistson the north edge of the Qinghai-Tibetan plateau [3]. During long-term evolution, the pikas evolved a series of physiological adaptations that allow them to thrive in the harsh environment and become a highly advanced hypoxia-tolerant mammal. Specifically, the pika obtained oxygen effectively from the hypoxic environment by larger superficial pulmonary alveoli and higher capillary density [4], thin walled pulmonary arterioles and blunted hypoxic pulmonary vasoconstriction (HPV) [5], an increase in erythrocyte count [6], reduction in the mean corpuscular volume [7], changes in hemoglobin (Hb) [8], and 2,3-diphosphoglycerate concentrations [5], and an increase in the oxygen affinity to Hb [8]; Secondly, a pika has a strong cardiac pumping function due to its larger heart and smaller weights of right-to-left ventricular plus septum [9]; Thirdly, a pika has a high oxygen utilization ratio by increasing the densities of capillary and mitochondrial [10], and myoglobin concentration in tissues [6,9]. In addition to these physiological mechanisms, a pika reduces dependence on oxygen by increasing anaerobic glycolysis in its skeletal muscle [11] and gluconeogenesis in liver [12]. The molecular mechanisms of these adaptations in pika have occurred due to a series of changes such as genetic evolution, the expression of tissue-specific proteins, and changes related to high altitude, including many functional cytokines as vascular endothelial growth factor (VEGF) [13,14], hemoglobin [3], HIF-1α [15,16], LDH-C_4_ [17], pyruvate carboxylase [12], myoglobin [9], inducible nitric oxide synthase (iNOS) [18], leptin [19,20] and cytochromec oxidase [21].

It is well known that LDH family enzymes catalyze the inter-conversion of pyruvate and lactate with the concomitant oxidation/reduction of the reduced form of nicotinamide adenine dinucleotide hydrogen (NADH) to nicotinamide adenine dinucleotide (NAD^+^) in the reactions [22]. Different forms of Lactate dehydrogenase (LDH) isozymes are comprised of expression products of three typical genes: *Ldh-a*, *Ldh-b*, and *Ldh-c*, which encode LDHA, LDHB and LDHC subunits, respectively [23,24]. LDH consists of A and B subunits that assemble into homo- or hetero- tetramers. These A and B subunits are distributed in various combinations in the body reflecting various energy metabolic requirements of different tissues, and are consistent with the catalytic properties of each isozyme [25,26]. However, the homo-tetramer LDH-C_4_ was previously detected only in testis and spermatozoa of mammals [27,28,29,30]. Surprisingly, our previous studies identified the expression of *Ldh-c* not only in testis and sperm, but also in somatic cells of plateau pika [17].

LDH-C_4_ has high thermostability [17], and its homolog from different species showed activity against longer carbon chain with α-hydroxy and α-keto acids than those of pyruvate and lactate [31,32,33,34,35,36,37,38,39,40,41], indicating that LDH-C_4_ has unique structural and functional properties [31,40,41,42]. In the present study, we compared the enzymatic kinetic characteristics among LDH-A_4_, LDH-B_4_, and LDH-C_4_ of plateau pika with the aim of exploring the pika’s adaptation mechanism to the hypoxic environment of the Qinghai-Tibet Plateau.

## 2. Results

### 2.1. Plasmid Construction and Recombinant Protein Expression

**Figure 1:**
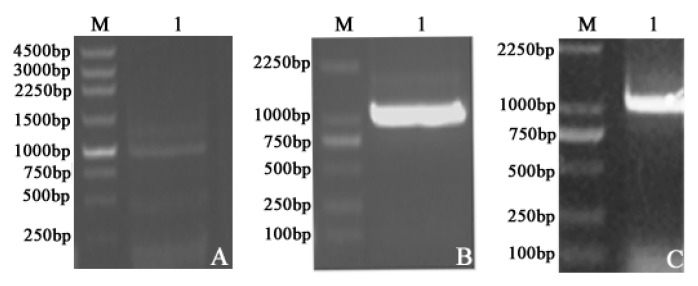
Amplification of plateau pika *Ldh-a, Ldh-b* and *Ldh-c.* Lane 1: PCR products of (**A**) *Ldh-a*; (**B**) *Ldh-b*; and (**C**) *Ldh-c.* M: DNA marker. The PCR fragments of *Ldh-a*, *Ldh-b*, and *Ldh-c* were 999 bp, 1005 bp and 999 bp. The sequence of transcripts was identical to that in the GenBank for *Ldh-a*, *Ldh-b*, and *Ldh-c*, suggesting that the expression plasmids pCold-SUMO*-Ldh-a*, pET-30a-*Ldh-b*, and pET-30a-*Ldh-c* encoding the full-length pika LDHA, LDHB and LDHC, respectively, were successfully constructed.

To study the enzyme kinetics of LDH-A_4_, LDH-B_4_, and LDH-C_4_ of plateau pika, the expression plasmids pCold-SUMO*-Ldh-a*, pET-30a-*Ldh-b*, and pET-30a-*Ldh-c*, respectively encoding the full-length pika LDHA, LDHB, and LDHC were constructed. In order to verify that the vectors contained the sequences of *Ldh-a*, *Ldh-b*, and *Ldh-c* and their correct expression, Polymerase Chain Reaction (PCR) of the plasmids and sodium dodecyl sulfate polyacrylamide gel electropheresis (SDS-PAGE) analysis of the expressed proteins were performed. Figure 1A–C show the agarose gel electrophoresis results of PCR products of *Ldh-a*, *Ldh-b*, and *Ldh-c*, respectively. Lane 1 in Figure 1A–C displayed clear and bright bands at approximately 1000 bp, matching the same number of base pairs of *Ldh-a* (999 bp), *Ldh-b* (1005 bp), and *Ldh-c* (999 bp) as our previous study [17]. By sequencing and alignment, the sequences of *Ldh-a*, *Ldh-b* and *Ldh-c* in the final plasmid constructs were determined to be 999 bp, 1005 bp and 999 bp in length and identical to those in the Genbank (HQ704676, HQ704677 and HQ704678, respectively).

**Figure 2:**
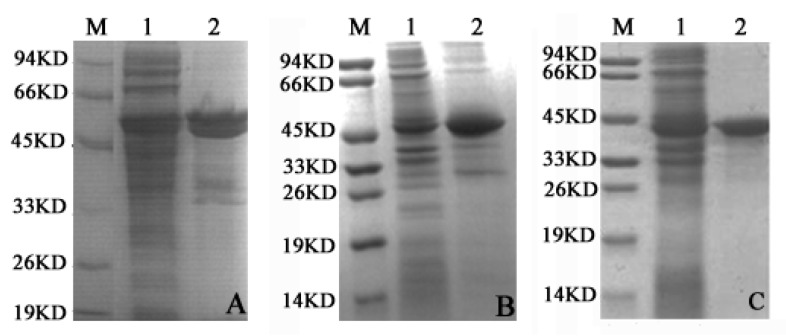
Analysis of the cell-free extracts recombinant protein by SDS-PAGE. (**A**) LDHA; (**B**) LDHB; and (**C**) LDHC. For (**A**–**C**) Lane 1 and Lane 2 represent the total protein of supernatant and precipitation, respectively, in the *E. coli* BL21 (DE3) cells; M: molecular weight standards. SDS-PAGE analysis indicates LDHA, LDHB and LDHC expression in supernatant and precipitate fractions.

The SDS-PAGE results of cell-free extracts (shown in Figure 2A–C) displayed the protein in the supernatant and precipitate fractions with dark bands around 40–50 kD. The recombinant LDH isoenzymes were expressed with His-tag (LDHA with His-tag and SUMO-tag) in the C-terminus, which was favorable to purification. These results demonstrated similar molecular weights to those of LDHA, LDHB, and LDHC, as we previously reported [17], indicating that expression plasmids pCold-SUMO*-Ldh-a*, pET-30a-*Ldh-b* and pET-30a-*Ldh-c* were successfully prepared, and expressed the proteins of interest correctly.

### 2.2. LDH-A_4_, LDH-B_4_ and LDH-C_4_ Purification and Activity Measurement

**Figure 3:**
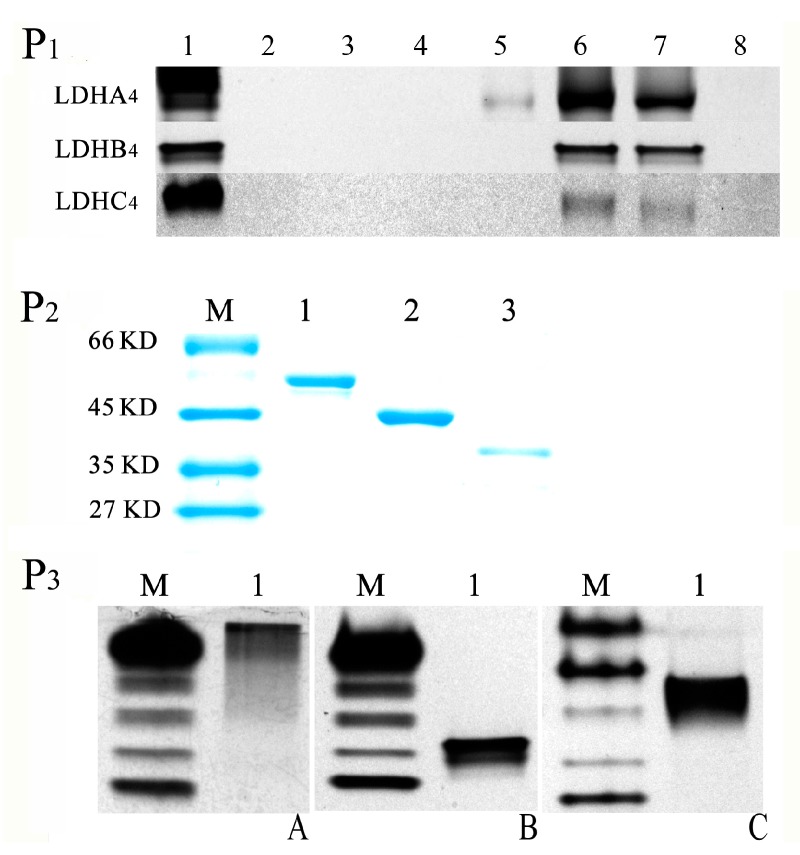
Purification and identification of LDH-A_4_, LDH-B_4_, and LDH-C_4_. (**P_1_**) Native PAGE of recombinant protein washed by imidazole at different concentrations. Lane 1: the supernatant before purification, Lane 2–7: collected elutions from Ni-NTA resins eluted with 10, 20, 50, 100, 250 and 500 mmol/L imidazole buffers, respectively. Lane 8: the remainder in the purified supernatant; (**P_2_**) SDS-PAGE analysis of the purified LDH-A_4_, LDH-B_4_, and LDH-C_4_. Lane 1–3 represented theLDH-A_4_, LDH-B_4_, and LDH-C_4_, respectively; M: marker; (**P_3_**) Identification of purified LDH-A_4_, LDH-B_4_, and LDH-C_4_. Lane 1 represents (**A**) LDH-A_4_; (**B**) LDH-B_4_, and (**C**) LDH-C_4_ by Native polyacrylamide gel electrophoresis (PAGE), respectively. M: marker was prepared by mixed kidney and skeletal muscle tissues of plateau pika.

**Figure 4:**
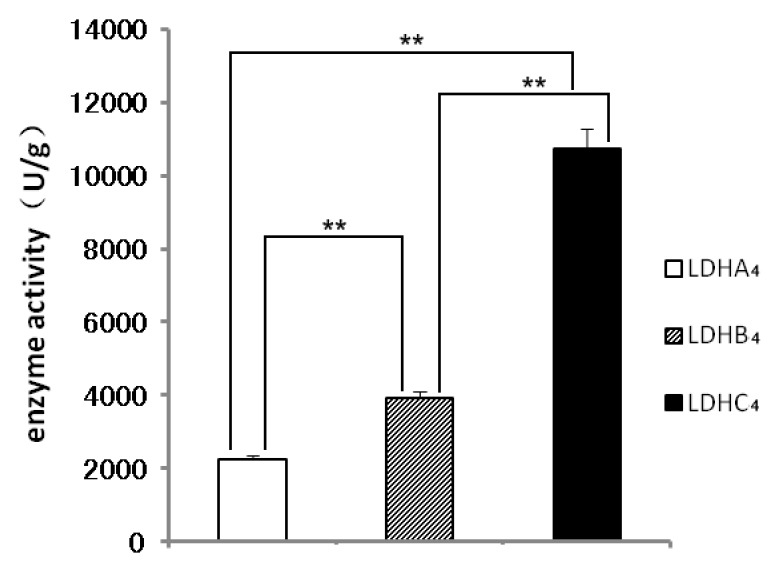
The enzyme activities of LDH-A_4_, LDH-B_4_ and LDH-C_4_. The enzyme activity of LDH-A_4_, LDH-B_4_, and LDH-C_4_ was 2241 ± 9.3 U/g protein, 3918 ± 37.9 U/g protein, 10741 ± 80.9 U/g protein, each sample size was 5, ** *p* < 0.01. The enzyme activity of LDH-C_4_ was significantly higher that of LDH-A_4_ and LDH-B_4_ (*p* < 0.01).

The native polyacrylamide gel electropheresis (native PAGE) results shown in Figure 3P_1_ indicate that LDH-A_4_ was observed in 100, 250 and 500 mmol/L imidazole buffer elutions with LDH activity, and LDH-B_4_ and LDH-C_4_ were observed in 250 and 500 mmol/L imidazole buffers elutions with LDH activity. LDH-A_4_, LDH-B_4_ and LDH-C_4_ bound so completely to Ni-NTA that no active protein was assayed in the remainder of the purified supernatant. As shown in Figure 3P_2_, the purified LDH-A_4_, LDH-B_4_, and LDH-C_4_, were represented with only one band as observed by SDS-PAGE, and the molecular weights were consistent with LDHA, LDHB, and LDHC, respectively. Figure 3P_3_ presents the LDH activity of the purified LDH-A_4_, LDH-B_4_, and LDH-C_4_ as determined by native PAGE. These results indicate that LDH-A_4_, LDH-B_4_, and LDH-C_4_ of plateau pika were successfully purified. As shown in Figure 4, the respective enzyme activity (*n* = 5) of LDH-A_4_, LDH-B_4_, and LDH-C_4_ was 2241 ± 9.3 U/g protein, 3918 ± 37.9 U/g protein, and 10,741 ± 80.9 U/g protein, and that of LDH-C_4_ was significantly higher than that of LDH-A_4_ and LDH-B_4_ (*p* < 0.01).

### 2.3. Enzyme Kinetics Properties of LDH-A_4_, LDH-B_4_, and LDH-C_4_

**Figure 5:**
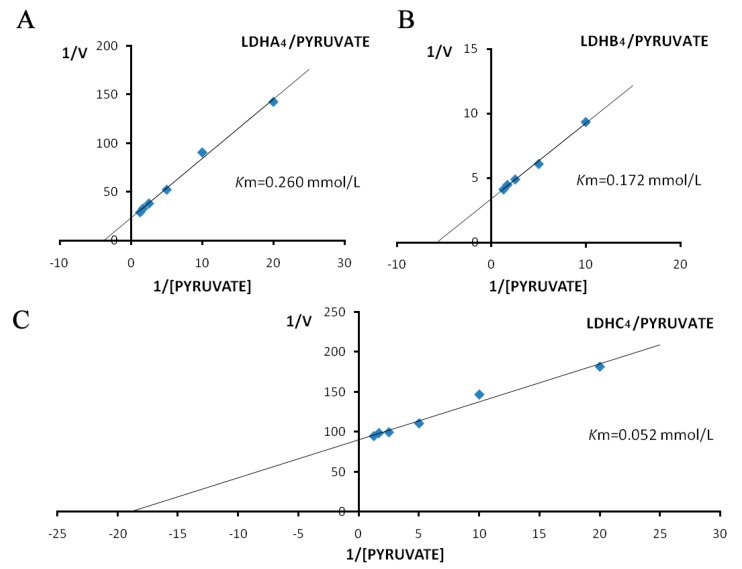
Double reciprocal plots by pyruvate as substrate of plateau pika LDH isozymes. Double reciprocal plots of (**A**) LDH-A_4_; (**B**) LDH-B_4_; and (**C**) LDH-C_4_, respectively. Reciprocal velocity (*V*) was calculated with the reciprocal Δ340 nm/min, plots were preformed by calculating the reciprocal reaction velocity *vs.* reciprocal pyruvate concentration at a constant concentration of NADH. The concentrations of pyruvate used were 0.05, 0.1, 0.2, 0.4, 0.6 and 0.8 mmol/L, respectively. NADH concentration was maintained at 0.15 mmol/L. The resulting *K*m of LDH-A_4_, LDH-B_4_, and LDH-C_4_ for pyruvate were 0.260, 0.172 and 0.052 mmol/L, respectively.

**Figure 6:**
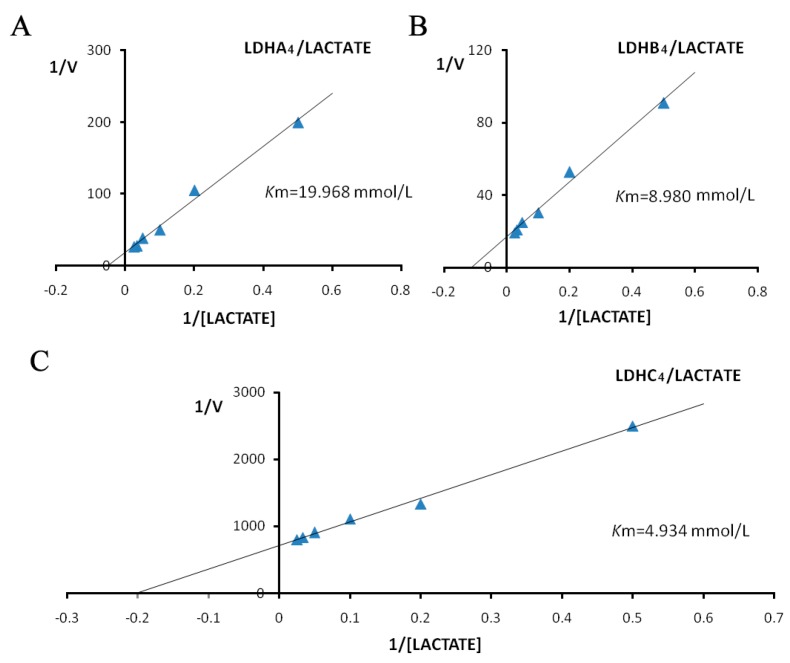
Double reciprocal plots by lactate as substrate of plateau pika LDH isozymes. Double reciprocal plots of (**A**) LDH-A_4_; (**B**) LDH-B_4_; and (**C**) LDH-C_4_, respectively. Reciprocal velocity (*V*) was calculated with the reciprocal Δ340 nm/min, plots were preformed by calculating the reciprocal reaction velocity *vs.* reciprocal pyruvate concentration at a constant concentration of NAD^+^. The concentrations of lactate used were 2, 5, 10, 20, 30 and 40 mmol/L. NAD^+^ concentration was maintained at 0.5 mmol/L. The resulting *K*m of LDH-A_4_, LDH-B_4_, and LDH-C_4_ for lactate were 19.968, 8.980, 4.934 mmol/L, respectively.

The *K*m values for LDH-A_4_, LDH-B_4_ and LDH-C_4_ were determined from Lineweaver-Burk double-reciprocal plots. The activities of the LDH isoenzymes in forward reaction (pyruvate + NADH + H^+^ → lactate + NAD^+^) increased with increasing concentration of the substrate pyruvate, as shown in Figure 5. The kinetic parameters of the forward reaction for LDH-A_4_, LDH-B_4_, and LDH-C_4_, calculated from the Lineweaver-Burk double-reciprocal plots of reciprocal pyruvate concentration *vs.* reciprocal velocity, had *K*m values of 0.260, 0.172, and 0.052 mmol/L, respectively. The activities of LDH isoenzymes in reverse reaction (lactate + NAD^+^ → pyruvate + NADH + H^+^) also increased with increasing concentration of the substrate lactate, as shown in Figure 6. The kinetic parameters of the reverse reaction for LDH-A_4_, LDH-B_4_, and LDH-C_4_, calculated from the Lineweaver-Burk double-reciprocal plots of reciprocal lactate concentration *vs.* reciprocal velocity, had *K*m values of 19.968, 8.980 and 4.934 mmol/L, respectively.

**Figure 7:**
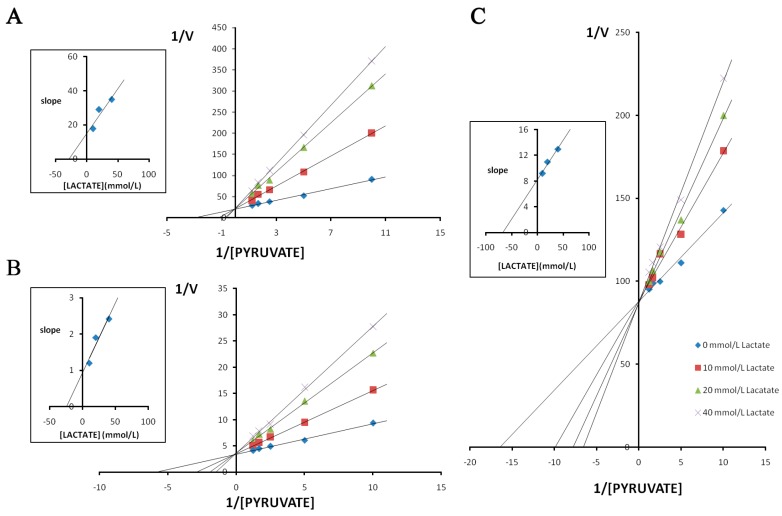
Effect of pyruvate on the inhibitory activity of lactate on plateau pika LDH isozymes. Double reciprocal plots of (**A**) LDH-A_4_; (**B**) LDH-B_4_; and (**C**) LDH-C_4_, respectively. Reciprocal velocity (*V*) was calculated with the reciprocal Δ340 nm/min, plots were preformed by calculating the reciprocal value of reaction velocity *vs.* reciprocal value of pyruvate concentration at a constant concentration of NADH. The concentrations of pyruvate used were 0.1 0.2, 0.4, 0.6 and 0.8 mmol/L. NADH concentration was maintained at 0.15 mmol/L. Upper left: determination of *K*i from plots of slope values against lactate concentrations. The resulting *K*i for pika LDH-A**_4_**, LDH-B**_4_**, LDH-C**_4_** by lactate were 25.9, 22.2 and 66.5 mmol/L, respectively.

**Figure 8:**
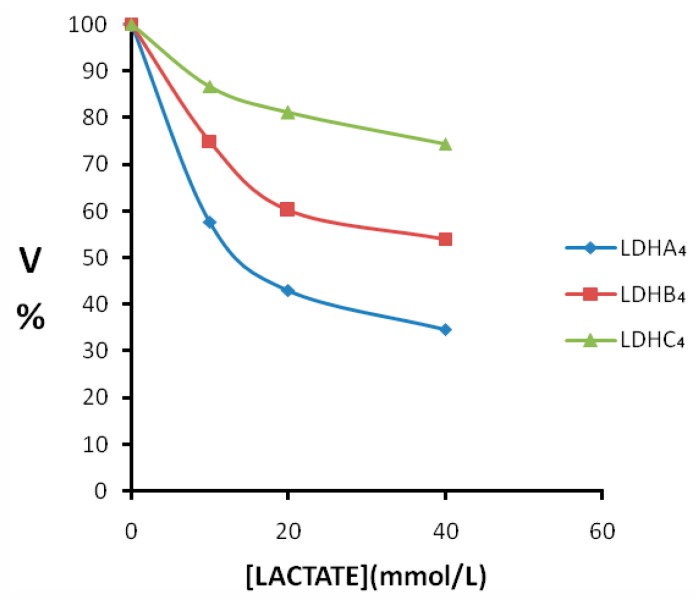
Effect of increasing concentration of lactate on plateau pika LDH isozymes. Pyruvate was used as substrate and lactate as an inhibitor. Velocities were calculated taking the maximum activity without the inhibitor lactate as 100%. Lactate was added at the concentration of 10, 20 and 40 mmol/L. The enzyme reaction velocity of LDH-C_4_ was less affected than that of LDH-A_4_ and LDH-B_4_ when the lactate concentrations were at 0–40 mmol/L.

The double reciprocal plots of initial velocities at different pyruvate concentrations and the inhibitory effect of lactate at different concentrations on pika LDH isozymes are shown in Figure 7. In all cases, the competitive inhibition was observed. Initial velocities were determined in the presence and absence of lactate of different concentrations. The *K*i values calculated from the Lineweaver-Burk double-reciprocal plots of reciprocal pyruvate concentration *vs.* reciprocal velocity. Reciprocals of initial velocity with substrate concentrations below the above-mentioned concentrations gave linear plots with these substrates. The results showed that the *K*i of lactate for LDH-C_4_ (*K*i = 65.500 mmol/L) was almost three times higher than that for LDH-A_4_ (*K*i = 26.900 mmol/L) and LDH-B_4_ (*K*i = 23.800 mmol/L). The inhibitory effects of lactate at different concentrations on the enzyme reaction velocity of LDH isozymes are shown in Figure 8. Under the experimental conditions employed in the present study, lactate at higher concentrations exhibited a strong inhibition of LDH-A_4_ and LDH-B_4_ activity, but only slightly inhibited LDH-C_4_ activity. The results indicated that LDH-C_4_ was less inhibited by lactate, which was likely beneficial in catalyzing the conversion of pyruvate to lactate even at high concentrations of lactate.

## 3. Discussion

LDH is the terminal enzyme of glycolysis with the role of reducing pyruvate to lactate with limiting oxygen [43]. LDH-C_4_ catalyzes the terminal reaction in the glycolytic pathway and displays a unique structure and functional properties [43,44].

Ultimately, the biochemical properties that distinguish LDH-C_4_ from the other LDH isoforms may be attributed to its high glycolytic rate. Studies have shown that since the same chemical reaction is being catalyzed, in spite of different kinetic constants, the equilibrium constant (*K*_eq_) is the same for all LDH isoenzymes, as stated in the Haldane equation [45,46]. The complex Haldane kinetics mechanism may be reduced to the Michaelis-Menten mechanism more tractably under special conditions of substrate-enzyme interactions [46]. Also, the basic Michaelis-Menten description can be modified in a logistical manner to fit the reaction parameters with *in vitro* conditions for the enzyme-catalyzed reactions [47]. In the sense it is even hyperbolic to logistic kinetics that kinetic constants may be considered as being the same, however with the amendment they would respectively correspond to various fractions from maximum velocity of outtake [45,47]. Based on this theory, the LDH isoenzyme pattern in itself may not change the equilibrium of the LDH reaction or influence the tissue lactate concentration [45]. However, the situation could be quite different during transition in energy metabolism where especially the glycolytic flux can undergo great rapid changes. During fast metabolic transitions involving significant pyruvate concentrations changes, primarily resulting from major changes glycolytic rate, the LDH isoenzyme pattern may be of great importance in the compound metabolic response to the altered energy metabolism [45].Therefore, we presumed that ATP was produced rapidly through anaerobic glycolysis by LDH-C_4_ in plateau pika, which stirs up the metabolic response to the changed energy metabolism, enhancing its adaptation to the hypoxic environment.

Previous studies have investigated in detail the enzymatic kinetics properties of LDH-C_4_ in other species [31,48,49]. The biochemical properties distinguishing LDH-C_4_ from the other LDH isoforms may be conducible to the high glycolytic rate. Compared with LDH-A_4_, pika LDH-C_4_ has a high *K*m for lactate (~2.0 mmol/L) and a low *K*m for pyruvate (~0.030 mmol/L) [31,48,49,50]. This finding suggests that LDH-C_4_ has a 60-fold higher affinity for pyruvate than that for lactate. The finding also implies that pyruvate conversion to lactate may occur even at high concentrations of extracellular or endogenous lactate [31,48,49,50]. This theory has been supported by an experiment that addition of excess lactate in the environment (50-fold excess in relation to pyruvate) did not affect adenosine triphosphate (ATP) production in capacitating spermatozoa [51]. In the present study, to investigate the enzyme kinetics of LDH-A_4_, LDH-B_4_, and LDH-C_4_ of plateau pika, the expression plasmids pCold-SUMO-*Ldh-a*, pET-30a-*Ldh-b*, and pET-30a-*Ldh-c*, encoding the full-length of pika LDHA, LDHB, and LDHC, were constructed and expressed. SDS-PAGE was used to determine the molecular weight and confirm the recombinant proteins. Figure 2A–C demonstrates that their molecular weights were about 50 kD, 40–45 kD and 40–45 kD for LDHA, LDHB, and LDHC, respectively. According to our previous paper [17], the open reading frames (ORF) of pika *Ldh-a*, *Ldh-b* and *Ldh-c* (accession numbers HQ704676, HQ704677, and HQ704678 in GenBank) were 999 bp, 1005 bp and 999 bp, respectively, encoding 332, 334 and 332 amino acids proteins, with their molecular weights of 36,557.5, 36,464.3 and 36,052.9 Da. Thus the molecular weight of the recombinant LDHA, LDHB, and LDHC in the current study (Figure 2A–C, Figure 3P_2_) was higher than the value calculated from their amino acid sequences deduced from the encoding cDNA. The addition of the His-tag resulted in a 5–10 kD deviation in the molecular weight of fusion proteins as shown in SDS-PAGE, and also indicated in other studies [52,53,54]. In addition, fusion proteins with the SUMO-tag added 12 kD to its molecular weight [55].

The recombinant expression shuttles (pET-30a-*Ldh-b*, pET-30a-*Ldh-c*, pCold-SUMO-*Ldh-a*) were transformed into *E*. *coli* BL21 cells and the enzymatic proteins (LDH-A_4_, LDH-B_4_, and LDH-C_4_) with functional activity were purified from protein fluid extracts. Our results indicate that the enzyme activity of the pika LDH-C_4_ was significantly higher than that of A_4_ and B_4_, that LDH-C_4_ was less sensitive to lactate inhibition than A_4_ and B_4_ (*p* < 0.01), and the *K*m values of LDH-C_4_ for pyruvate and lactate were also higher than that of A_4_ and B_4_. Compared with LDH-A_4_ and LDH-B_4_, LDH-C_4_ had a low *K*m for pyruvate (~0.052 mmol/L) and a high *K*m for lactate (~4.934 mmol/L); and the affinity of LDH-C_4_ for pyruvate is 90-fold higher than that for lactate. These properties of pika LDH-C_4_ were beneficial to catalyze the conversion of pyruvate to lactate even at high concentration of lactate.

Although the conversion mediated by LDH does not generate ATP, the reaction depends on NADH as a cofactor that accompanies oxidation to NAD^+^; the concentration of NAD^+^ is a rate-limiting factor in glycolysis and necessary for continued glycolysis [56]. Previous studies demonstrated that knockout of *Ldh-c* or inhibition of LDH-C_4_ in sperm led to rapid decline in sperm ATP levels [56,57], a decrease in progressive motility, and a failure to develop hyper-activated motility. It was revealed that all consumed^13^C-labeled pyruvate added in sperm culture medium was converted to lactate rather than oxidized in the tricarboxylic acid (TCA) cycle in metabolic tracing experiments; in the presence of exogenous pyruvate, the ATP concentration was increased by more than 50% [51]. When carbonyl cyanide m-chlorophenylhydrazone (CCCP) and NaCN was applied to inhibit the oxidative phosphorylation in mitochondria, the amount of ATP was kept at the equivalent level to that without CCCP and the vigorous motility of sperm was maintained [51,58]. These results suggest that LDH-C_4_ is essential in sperm glycolysis which has an important role in providing abundant ATP for sperm motility [56,58,59], and it is related to LDH-C_4_ enzymatic kinetics properties.

Plateau pika has a strong adaptability to the hypoxic plateau environment of the Qinghai-Tibet Plateau, given the sperm-specific lactate dehydrogenase (*Ldh-c*) gene is also expressed in their somatic cells. In light of the enzymatic kinetics properties, we propose that the pika could reduce dependence on oxygen and enhance the adaptation to the hypoxic environments due to increased anaerobic glycolysis by LDH-C_4_.

## 4. Materials and Methods

### 4.1. Reagents and Animal Procedures

All reagents were from Sangon (Sangon, Shanghai, China) unless otherwise noted. Plateau pikas were live-trapped from Haibei Alpine Meadow Ecosystem Research Station at an altitude of 3800 m in Qinghai Province, China. All animals were first anesthetized with sodium pentobarbital (5%), and then sacrificed by cervical dislocation immediately before dissection. Skeletal muscle, kidney, and testis were rapidly removed and frozen in liquid nitrogen for long-term storage. All procedures involved in the handling and care of animals were approved by the China Zoological Society according to the China Practice for the Care and Use of Laboratory Animals (permit number: GB 14923-2010).

### 4.2. Expression Plasmids Construction

In our previous study [17], *Ldh-a*, *Ldh-b*, and *Ldh-c* of plateau pika testis were cloned and deposited in GenBank with the accession numbers HQ704676, HQ704677, and HQ704678, respectively. Three lengths of *Bam*HI*/Xho*I fragments (999 bp, 1005 bp, 999 bp representing *Ldh-a*, *Ldh-b*, and *Ldh-c* coding sequences, respectively) were PCR amplified from plateau pika testis cDNA using Premix Ex Taq Version Kit (Takara, Kyoto, Japan). The PCR primers for *Ldh-a* were 5′-CGGAATTCATGGCAGCTCTCAAGGATCAG-3′ (sense) and 5′-CCGCTCGAGGAACTGCAGCTCCTTCTGGAT-3′ (antisense), for *Ldh-b* were 5′-CGGAATTCATGGCAACCCTGAAGGAAAAACTCAT (sense) and 5′-CCGCTCGAGCAGGTCCTTCAGGTCCTTCTGGA-3′ (antisense), and for *Ldh-c* were 5′-CGGGATCCATGTCGACAGTCAAGGAGC-3′ (sense) and 5′-CCGCTCGAGAAACACCAGGTCCTTCTGGAC-3′ (antisense), respectively. PCR conditions were 5 min at 95 °C, 30 cycles of 45 s at 95 °C, 45 s at 65 °C, 1 min at 72 °C, and a final elongation step at 72 °C for 10 min. PCR products were determined with agarose gel electrophoresis. Subsequently, the *Bam*HI*/Xho*I fragment, *Ldh-a* was inserted into pCold-SUMO expression vector (BPI, Beijing, China); the other two fragments, *Ldh-b* and *Ldh-c* were inserted into pET-30a (+) expression vector (Novagen, Darmstadt, Germany). The final plasmid constructs of pCold-SUMO-*Ldh-a*, pET-30a-*Ldh-b*, and pET-30a-*Ldh-c* had been sequenced in BGI (Beijing Genomics Institute, Beijing, China).The recombinant expression shuttles (pCold-SUMO*-Ldh-a*, pET-30a-*Ldh-b*, and pET-30a-*Ldh-c*) with six His-tags in the C-terminus were transformed into *Escherichia coli* (*E. coli*) BL21 (DE3) cells.

### 4.3. Protein Expression, Purification, Analysis and Enzyme Activity Measurement

*E. coli* BL21 (DE3) cells were cultured in media of Lysogeny broth (LB). *E. coli* clones with plasmids (pCold-SUMO*-Ldh-a*, pET-30a-*Ldh-b*, and pET-30a-*Ldh-c*) were inoculated into a liter of medium with 100 μg/mL ampicillin or 50 μg/mL kanamycin and cultured at 37 °C. When *A*600 value arrived at 0.6, isopropyl-β-d-thiogalactopyranoside (IPTG) reagent was added till the final concentration of 1 mmol/L, followed by further culturing at 25 °C for 10 h to induce the expression of *Ldh-a*, *Ldh-b*, and *Ldh-c*. To determine LDHA, LDHB and LDHC expression in the supernatant and precipitate of *E. coli* cells, bacterial cells were collected by centrifugation and resuspended in 20 mmol/L phosphate buffer saline (PBS) (pH 8.0), and disrupted by ultrasonication (36 × 5 s pulses with 5 s intervals). The supernatant was then collected; the precipitate was resuspended and washed in 20 mmol/L PBS (pH 8.0) with 8 mol/L urea. SDS-PAGE was performed to assay the collected supernatant and treated precipitate with a steady current voltage electrophoresis instrument (Beijing Liuyi Instrument Factory, Beijing, China) as follows: 6 μL samples were loaded on a 12% (*w/v*) separating gel with a 5% (*w/v*) stacking gel in Tris*-*glycine buffer (pH = 8.3). The electric voltage was 80 V in the stacking gel and 120 V in the separating gel. The gel was stained in with Coomassie staining solution (10% methyl alcohol, 10% acetic acid, 0.2% R-250) for 4 h, and finally stored in the destaining solution (10% methyl alcohol, 10% acetic acid).

To obtain the active enzyme protein in the supernatant of *E. coli* cells, bacterial cells were collected with following procedures: 4000 r/min centrifugation (15 min), resuspension in 20 mmol/L PBS (pH = 7.0), and disruption by repeated freeze-thaw cycles (in liquid nitrogen and 37 °C water bath) for 10 times. In freeze-thaw cycles, Triton X-100 (final concentration of 1%), lysozyme (final concentration of 1%) and DNA enzyme (final concentration of 0.2%) were added. The lysates were centrifuged at 15,000 r/min under a temperature of 4 °C for 10 min, and the obtained supernatant was used for purification. Ni-NTA resin (QIAGEN, Hilden, Germany) was used to purify the recombinant LDHs. The resins were washed twice with increasing concentrations of imidazole at 10, 20, 50, 100, 250 and 500 mmol/L in PBS gradient (50 mmol/L NaH_2_PO_4_, 300 mmol/L NaCl, pH = 8.0). Each fraction was collected using individual collection tubes and detected by native PAGE. Elutions with LDH activity were totally collected. Enzyme liquids were obtained from elutions after removing imidazole by ultrafiltration (Ultrafiltration Tubes, 50 kD, 15 mL, Merck Millipore, Billerica, MA, USA). On average, a final amount of 3 mg LDH isoenzymes were purified from every liter of bacterial culture following this approach. LDH isoenzymes stocks were finally stored in PBS (pH = 8.0) at 4 °C until following steps. The LDH isoenzymes were prepared simultaneously under the same conditions [60].

SDS-PAGE was used to detect the protein purification and native PAGE was used to test and verify the enzyme activity. Native PAGE experiment was also performed with a voltage electrophoresis apparatus (Beijing Liuyi Instrument Factory, Beijing, China) with the following conditions: 8% (*w/v*) separating gel with a 4% (*w/v*) stacking gel (non-denaturing polyacrylamide gel) in Tris-glycine (pH = 8.3), and 6 μL samples were loaded. LDH bands were stained at 37 °C in a specific staining reagent (a mixture of 0.5 mol/L phosphate buffer (pH = 7.5), 10 mL of 1 mg/mL nitrobenzenethiocyanate chloride (NBT), 4 mL of 5 mg/mL NAD^+^, 2.5 mL of 1 mol/L sodium lactate, 2.5 mL of 0.1 mol/L NaCl and 1 mL of 1 mg/mL phenazinemethosulfate (PMS)) for half an hour in the dark. The stained gels were then rinsed with ddH_2_O and stored in the storage liquid (10% glycerol and 7% acetic acid). The reference marker for native PAGE was made with a mixture of pika kidney and skeletal muscle, tissues were homogenized on ice as a 1:4 (*w*/*v*) dilution in 0.9% physiological saline, and centrifuged at 4000 r/min at 4 °C for 10 min, and finally the supernatant was collected.

Protein concentration was determined by Bradford assay (Bradford protein assay kit, Nanjing Jiancheng Biotech Inc., Nanjing, China). Enzyme activity was measured by Bergmeyer assay (Lactate dehydrogenase kit, Nanjing Jiancheng Biotech Inc., Nanjing, China).

### 4.4. LDH-A_4_, LDH-B_4_ and LDH-C_4_ Kinetics Properties

The activity of LDH was measured using UV spectrophotometer (Unicon 2800, Shanghai, China) by recording the change of 340 nm absorbance produced by NADH oxidation. Assays were kept at consistent temperature of 37 °C. Calculation of *K*m value for pyruvate and lactate was resulted from Lineweaver-Burk plots. *K*i value of lactate was determined from *K*m and *V*max*,* which was obtained with or without inhibitor added to reaction buffer via various concentrations of pyruvate at a constant inhibitor concentration, followed by plotting the slope (*K*m*/V*max value) against inhibitor concentrations. The mixed reagent of reaction to from pyruvate lactate contained 0.15 mmol/L NADH, 50 mmol/L PBS (pH8.0) and the sodium pyruvate gradients as substrate were performed at 0.05, 0.1, 0.2, 0.4, 0.6 and 0.8 mmol/L for LDH-A_4_, LDH-B_4_, and LDH-C_4_, and the enzyme persisted at a constant inhibitor concentration. The reaction from lactate to pyruvate contained 0.5 mmol/L NAD^+^, 50 mmol/LPBS, (pH 8.0); sodium lactate gradients as substrate were performed at 2, 5, 10, 20, 30 and 40 mmol/L for LDH-A_4_, LDH-B_4_, and LDH-C_4_. The concentration gradients of sodium lactate as inhibitor were added as 10, 20, and 40 mmol/L, respectively; and the substrates, coenzymes, and other buffers followed the method of the reaction from pyruvate to lactate as outlined above. A Δ*E*_340_ value of 0.06–0.07 per minute was provided in the experiment. The enzyme preparation was diluted with PBS (pH = 8.0) and assayed in a 1 cm light path. Before starting the reaction by addition of substrate, the coenzyme was incubated with the buffer used in the assay for 10 min at 37 °C. In total, 3 mL volumes of reagents were added in the process [61]. All chemicals were analytically pure from Sangon (Sangon, Shanghai, China). All measurements in the study were repeated 5 times for the average values.

### 4.5. Statistics

The data are presented as mean ± standard deviation (SD). Statistical analysis was performed by one-way analysis of variance (ANOVA) followed by Duncan’s test using SPSS 22.0 (SPSS Inc., Chicago, IL, USA). Values of *p* < 0.01 was considered highly significant and *p* < 0.05 was considered statistically significant.

## 5. Conclusions

In light of the enzymatic kinetics properties, we suggest that the pika could reduce dependence on oxygen and enhance adaptation to the hypoxic environment due to its increasing anaerobic glycolysis to produce ATP rapidly by LDH-C_4,_ which is often the role of LDH-A_4_ in most mammal species.
